# The Application of Next Generation Sequencing in DNA Methylation Analysis

**DOI:** 10.3390/genes1010085

**Published:** 2010-06-04

**Authors:** Yingying Zhang, Albert Jeltsch

**Affiliations:** School of Engineering and Science, Jacobs University Bremen, Campus Ring 1, D-28759 Bremen, Germany; E-Mail: y.zhang@jacobs-university.de

**Keywords:** DNA methylation, next generation sequencing, bisulfite conversion, methylome

## Abstract

DNA methylation is a major form of epigenetic modification and plays essential roles in physiology and disease processes. In the human genome, about 80% of cytosines in the 56 million CpG sites are methylated to 5-methylcytosines. The methylation pattern of DNA is highly variable among cells types and developmental stages and influenced by disease processes and genetic factors, which brings considerable theoretical and technological challenges for its comprehensive mapping. Recently various high-throughput approaches based on bisulfite conversion combined with next generation sequencing have been developed and applied for the genome wide analysis of DNA methylation. These methods provide single base pair resolution, quantitative DNA methylation data with genome wide coverage. We review these methods here and discuss some technical points of special interest like the sequence depth necessary to reach conclusions, the identification of clonal DNA amplification after bisulfite conversion and the detection of non-CpG methylation. Future application of these methods will greatly facilitate the profiling of the DNA methylation in the genomes of different species, individuals and cell types under healthy and disease states.

## 1. Introduction

Multicellular organisms are composed of various cell types which contain the same genetic information but display different phenotypes. Cellular differentiation is orchestrated by epigenetic processes, which control the packaging and function of chromatin and regulate gene expression in a heritable fashion without changing the DNA sequence [1,2]. These epigenetic processes constitute a link between genotype, environment, phenotype and disease [3,4,5,6,7]. DNA methylation is a major form of epigenetic modifications and is the most common covalent modification of DNA in eukaryotes [8,9,10]. Deciphering the genome wide DNA methylation profile is critical for the understanding of the biological role of DNA methylation and the correlation of DNA methylation with other epigenetic mechanisms. Recently various high-throughput approaches based on Next Generation Sequencing (NGS) have been developed and applied in combination with bisulfite conversion of the DNA for the genome wide DNA methylation analysis in mammals and plants [11]. This review will focus on the application of different next generation sequencing methods for DNA methylation analysis in eukaryotes, discuss the merits and limitations of these methods and deal with some special features like the sequence depth necessary to draw conclusions on DNA methylation, methods to identify and exclude clonal DNA amplification during the procedure and approaches to detect non-CpG methylation.

DNA methylation refers to the covalent addition of a methyl group from *S*-adenosyl-*L*-methionine to the nucleotide bases, which occurs at the C-5 atom of cytosines in eukaryotes. DNA methylation is catalyzed by DNA methyltransferases, which are responsible to establish the DNA methylation pattern in early development and maintain it during cell division [8]. In mammals, cytosine methylation mainly happens in CpG dinucleotides in a cell type specific pattern. The CpG dinucleotides are notably under-represented and distributed unevenly in mammalian genomes. Since methylated cytosine is mutagenic, it has a tendency to get lost during evolution leading to a genome wide depletion of CpG sequences. Clusters of unmethylated CpG sites are not affected and form characteristic CpG islands (CGIs) in the genome [12] which cover around 0.68 % of the genome, but contain 6.8% of all CpG sites [13]. They coincide with promoter regions of approximate 70% of all human genes [14]. The CGIs are usually unmethylated in germline and in differentiated cells, although they may be subject to tissue specific gain of methylation (see below). The methylation of promoter-related CGIs causes gene silencing. Therefore the study of the methylation state of CGIs is one of the focuses of DNA methylation analysis. The majority of the CpG sites outside of CGIs are methylated. In plants, DNA methylation can occur on cytosine in any sequence context, including the symmetrical CpG, CHG sequences and asymmetric CHH sequences (with H = A, T or G) and non-CpG methylation has recently been reported in mammals as well [15,16].

DNA methylation plays essential roles in mammals. Together with other epigenetic mechanisms like histone modifications, and non-coding RNAs, DNA methylation can stably alter the gene expression pattern in cells, which should happen at proper time and proper place during development and cell differentiation. In addition, DNA methylation also contributes to the condensed, repressive state of chromatin, the maintenance of the genome stability, and the parental origin dependent allele-specific gene silencing in imprinted loci and the X chromosome inactivation in females [6,8,17]. Erroneous DNA methylation leads to an aberrant expression of genes, genome instability, and contributes to the development of cancer, aging and the etiology of complex multifactorial diseases [3,4,18,19,20].

**Figure 1:**
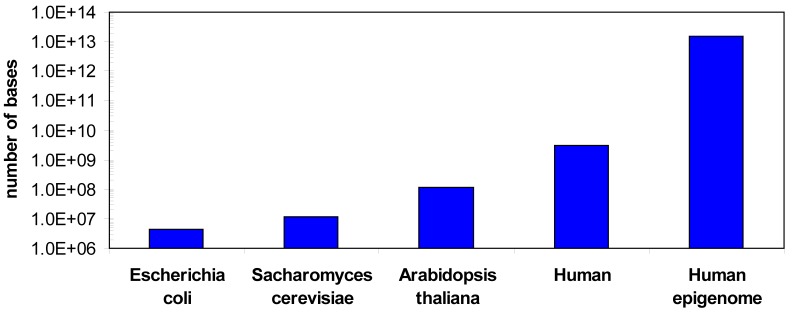
Comparison of the sizes of different genomes and the human epigenome. A minimum epigenome size was estimated considering 100 different cell types and 50 relevant developmental or disease specific states.

One of the challenges in DNA methylation analysis is that although there is only one genome for each organism, there can be hundreds of epigenomes, because the DNA methylation changes with cell type and during development or disease processes and sometimes in response to environment. For example, in the human methylome cell type and developmental specific changes in the methylation pattern [15,21,22,23,24,25,26], changes in the ratio of non-CpG and CpG methylation [15,16] or different methylation states of different gene copies in the same cell [27,28] have been observed. In addition, DNA methylation can also be variable among certain individuals, even between twins [28,29,30]. Hence the human epigenome is much larger than the genome (Figure 1). In addition, for bisulfite sequencing DNA methylation analyses several sequencing reads are required for each base to determine its methylation state, which further increases the sequencing demands (see below).

The DNA methylation information can so far not be read out by routine direct sequencing or by hybridization-based methods and it is erased by polymerase chain reaction (PCR) and cloning of the DNA. Recently various technologies for the genome-wide DNA methylation analysis have been developed as summarized in [9,11,31]. These technologies are based on three approaches to discriminate the methylated and unmethylated cytosines.

**Figure 2:**
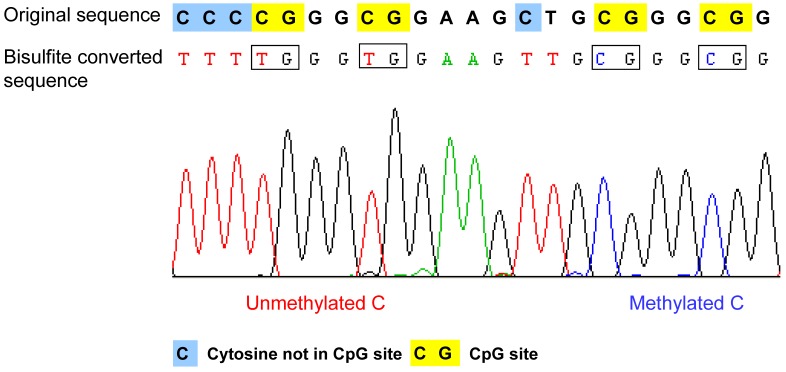
Example of the bisulfite sequencing result of a single read. After bisulfite conversion and the following amplication step, the unmethylated cytosines are converted to thymines, and the methylated cytosines remain as cytosine. Therefore, the methylated cytosine and unmethylated cytosine can be distinguished according to the sequencing result. Original sequence: DNA sequence before bisulfite treatment.

1. Methylation sensitive restriction enzyme digestion. The genomic DNA can be digested by methylation sensitive restriction enzymes like *Hpa*II and McrBC to discriminate and/or enrich methylated or unmethylated DNA. The methods based on this approach are limited by providing methylation data only at the restriction enzyme recognition sites or adjacent regions.
2. Affinity purification. The methylated or unmethylated fractions of genomic DNA can be immunoprecipitated by using antibodies against methylated cytosine, methyl-CpG binding domains or other protein domains [32,33,34,35,36]. Using this method, the genome coverage is limited by the composition of the array for hybridization, and the distribution of the potential affinity targets in the genome, e.g. the density of methylated cytosines or CpG sites, which are unevenly distributed in the genome. The exact methylation state of individual CpG sites cannot be determined using this approach.
3. Bisulfite conversion of DNA. The method is based on the selective deamination of cytosine but not 5-methylcytosine by treatment with sodium bisulfite [37,38]. Briefly, in the presence of sodium bisulfite, all the unmethylated cytosines are chemically converted to uracil, which is amplified as thymine during PCR. In contrast, the methylated cytosines are not converted, such that in the final sequencing result, the 5-methylcytosine will be still detected as cytosine. Therefore, after bisulfite conversion, the methylated and unmethylated cytosines can be distinguished according to the sequence changes (Figure 2). The bisulfite conversion efficiency is critical for the accuracy and the reliability of the results, especially for non-CpG methylation analysis. The incomplete conversion of unmethylated cytosine to uracil or inappropriate conversion of methylcytosine to thymine can cause over- or underestimination of the methylation level [39,40]. It is also noteworthy that the bisulfite conversion technique cannot be used to discriminate the methylated cytosine from 5-hydroxymethylcytosine (5hmC), which has been recently detected in the Purkinje neurons and embryonic stem cells [41,42]. The underlying reason is that after bisulfite conversion, 5hmC is not deaminated to thymine, but converted to cytosine 5-methylenesulfonate, which is read as cytosine during Sanger sequencing [43,44].

Following one of these methylation-specific genomic DNA pre-treatments, various methods have been used to read out the DNA methylation information. PCR, gel electrophoresis, southern blotting, mass spectrometry and pyrosequencing have been developed and applied for specific loci or low throughput methylation analysis. For global DNA methylation profiling, the methods developed in the past decade can be classified into three stages [11,31]. The first stage was based on gel electrophoresis. The Restriction Landmark Genomic Scanning method which is based on methylation sensitive restriction enzyme digestion and two dimensional electrophoresis, was the first method applied for genome wide DNA methylation analysis [45]. The second stage was based on the application of microarrays. This technology provided an important platform for DNA methylation profiling and it had been widely used in combination with all the above mentioned methylation dependent DNA pretreatment methods [32,33,34,35,36,46]. However, the limited genome coverage, potential cross-hybridization and competitive hybridization, the difficulties in the signal normalization, data quantification and the low to moderate resolution of the dataset, impede the further application of this method [47]. With the available NGS methods, nowadays DNA methylation analysis has come to a third stage – sequencing based methylation profiling.

After bisulfite conversion, the methylation state of the DNA can be determined by DNA sequencing [37,38], methylation specific PCR [48], or restriction digestion [49]. Comparing with other methods, the sequencing of subcloned individual DNA molecules from bisulfite converted DNA provides the most reliable and detailed information on the methylation pattern for every single CpG site and it has been regarded as the “gold” standard of DNA methylation analysis for a long time. Furthermore, it provides unambiguous methylation information for haplotypes of DNA molecules in a qualitative and quantitative manner. In addition, for a real genome wide DNA methylation analysis, bisulfite conversion in combination with sequencing is the best choice out of the available methods, because bisulfite conversion can be done for the whole genomic DNA, which is not limited by the presence of certain restriction enzymes recognition sites or the high CpG density.

In the past, the bisulfite sequencing method has been often used for specific loci or for the confirmation of the accuracy of the newly developed DNA methylation analysis methods. For example, we used this method previously and analyzed the promoter methylation state of 190 genes on human chromosome 21, and measured the methylation difference of 16 amplicons among 20 individuals [22,28]. The high resolution of the dataset provided the opportunity to observe the methylation difference between alleles in the non-imprinting region of human autosomes, which was rarely reported before. The fast development of NGS methods, which can generate millions of reads each corresponding to the sequence of a single DNA molecule in one run without subcloning, has brought new opportunities to the wide usage of the bisulfite sequencing method for genome-wide DNA methylation analysis.

## 2. Application of next generation sequencing methods for DNA methylation analyses

Recently, several NGS platforms have been developed by different companies, including 1) 454 sequencing, from Roche Applied Science, 2) Illumina Genome analyzer (Solexa sequencing), from Illumina, 3) SOLiD^TM^ (Supported Oligonucleotide Ligation and Detection sequencing), from Applied Biosystems, 4) HeliScope Single Molecular Sequencer, from Helicos BioSciences. These methods share some common technological features: the template DNA is immobilized to a solid surface or support, such that the sequencing for the clonally amplified or single DNA molecule templates can be performed in parallel, and thousands to billions of sequence reads can be obtained in a single run [50,51]. The technological improvements dramatically decreased the sequencing costs per base and make it possible to generate genome wide bisulfite sequencing methylation data at single base resolution in a short time.

### 2.1. sequencing based DNA methylation analysis

Among the different platforms, 454 sequencing was the first commercially available NGS method. It is based on emulsion PCR for template preparation and pyrosequencing. Comparing with other available NGS platforms, the read length from 454 sequencing is the longest. Currently, the average read length is around 330 bp [51]. The long sequence reads provide important advantages for DNA methylation analysis: 1) they include many CpG sites, such that complex methylation patterns of individual DNA molecules can be determined. 2) The longer reads can be easier and more accurately aligned to the reference sequence, especially in repetitive regions of the genome. 3) The long reads have bigger chance to cover more genotype information like single nucleotide polymorphisms (SNPs) in the neighborhood of cytosines, making it possible to analyze the correlation between DNA methylation and genotype, which is a phenomenon just becoming to be realized [27,28,52]. Disadvantages of the method are the relatively high sequencing cost when compared with to other NGS methods, and higher error rates in calling homopolymeric stretches of identical bases, which happen quite often in bisulfite converted DNA.

In combination with bisulfite conversion, 454 sequencing has been used to analyze the methylation state of more than 100 PCR products amplified from different tissues in a single run [53]. For each PCR product, more than 1600 individual sequences were generated. The method was also used to analyze the methylation state of CGIs in human blood cells and sperm DNA samples [54], and methylation patterns of four genomic regions in the breast cancer tissues and sera from more than 50 individuals [55]. However, so far 454 sequencing has not yet been widely used for genome wide DNA methylation analysis.

### 2.2. Illumina Solexa sequencing based DNA methylation analysis

The Illumina Genome Analyzer was the first short read sequencing platform. The amplification of the templates from single molecules is conducted *in situ* via bridge amplification and the sequencing is based on sequencing-by-synthesis technology that employs reversible terminators with removable fluorescent dyes. Comparing this method to 454 sequencing, the read length is shorter, which ranges from 35 bp to 2x75 bp. However, the throughput of Illumina Solexa sequencing is higher, therefore the average cost per base is lower than in 454 sequencing. Both methods were compared in detail in a recent review [51]. So far, Illumina sequencing technology is the most widely used method for DNA methylation analysis both in genome wide level and also in targeted regions.

For genome wide DNA methylation analysis, the first single base resolution methylome profiles from *Arabidopsis thaliana* were obtained by two groups using the Illumina 1G Genome Analyzer and Solexa sequencing technology in combination with bisulfite conversion of genomic DNA [56,57]. The genome coverage of the datasets and the average number of reads for single locus (read depth) were slightly different between these two works. Cokus *et al.* reported that 2.6 billion nucleotides were mapped to the unique genomic locations, which covered around 86% of the 43 million cytosines in the 119Mb *Arabidopsis* genome [56]. On average, the read depth was around 20. Lister *et al.* reported that around 39 million unique and non-clonal reads covering 78.5% of the cytosines in the genome with at least two reads. The average read depth was 8 per base for each DNA strand [57]. One year later, Lister *et al.* employed a similar strategy and presented the first human methylome at single-base resolution for human stem cells and fetal lung fibroblasts [15]. This was a considerable achievement since with its 3.08 Gb the human genome is about 30 times larger than the *Arabidopsis thaliana* genome (119 Mb). In each cell type, over 86% of both strands of the human reference sequence were covered by at least one read, accounting for 94% of the cytosines in the genome. The average read depth in each cell type was around 14.5 per strand. In this analysis, a large fraction (24.5%) of non-CpG methylation in human stem cells was detected for the first time. Recently, Laurent *et al.* reported dynamic DNA methylation changes in human stem cells and differentiated cells using bisulfite conversion and sequencing by Illumina Genome Analyzer II [16]. In each cell type, on average 400 million reads were mapped uniquely to the reference genome, which covered >60% of cytosines in the genome with at least 3 reads. They observed non-CpG methylation not only in human stem cells (～20%), but also in primary fibroblast cells (～15%) and monocytes (～8%).

Illumina Solexa sequencing has also been widely used for non-genome wide DNA methylation analysis. As mentioned above, DNA methylation is highly variable between cell types, developmental stages and disease states. Currently, the high cost for sequencing-based genome wide DNA methylation analysis, does not allow to perform genome-wide DNA methylation analysis for multiple cell types in parallel. This has lead to approaches to analyze the DNA methylation state in many samples in a reduced part of the genome, in specific target regions or at lower sequence depth.

1) The reduced representation bisulfite sequencing (RRBS) method was developed and employed to map the methylation status of the murine genome in different cell lines [21]. The principle of the method is to reduce the complexity of genomic DNA by digesting the genomic DNA into small fragments using methylation insensitive restrictive enzymes like *Msp*I, which recognizes CCGG sequence that are enriched in CGIs. After size selection for short fragments, the digested DNA was bisulfite converted and sequenced by Illumina Genome Analyzer. The library generated from *Msp*I digestion was predicted to contain nearly 90% of CGIs in the mouse genome [58]. Recently, this method has been adapted for the DNA methylation profiling in the human genome, especially for the identification of the methylation changes in human clinical samples based on small amounts (30 ng) of genomic DNA [59]. The genomic coverage is not limited to the CGI (50%) and gene core promoters (65%), but other regions e.g. exons, 3’untranslated regions and repetitive elements are included as well [59].

2) Two similar approaches based on restriction enzymes digestion and Illumina sequencing, but without bisulfite conversion, have been developed for human methylome analysis. One is called Methyl-sensitive cut counting (MSCC) [60]. Here, the methylation sensitive restriction enzyme *Hpa*II, which cuts unmethylated CCGG sequence, is used to digest the genomic DNA and the generated library is sequenced by Illumina sequencing to reveal unmethylated sites [60]. Another similar approach is called methyl-sequencing [61]. Here, the isoschizomers *Hpa*II and *Msp*I are both used to digest the genomic DNA. After adaptor ligation and size selection, Illumina sequencing is used to sequence the library. As *Hpa*II only digest the unmethylated sites and *Msp*I can digest the sites regardless of methylation, the methylation state of single CpG site can be determined by comparing the different reads number from the two libraries [61]. These methods are useful in reducing the complexity of genomic DNA by focusing on the CpG sites in specific sequence context, but on the other hand they cannot provide a methylation map at single base pair resolution.

3) Based on Illumina sequencing and bisulfite conversion, array capture [62] and Padlock capture [60,63] were developed for the target specific DNA methylation analysis. Hodges *et al.* developed a method called bisulfite capture based on hybrid selection techniques. An array containing the probes designed to be complementary to the sequence of interest is used to enrich the target sequences from bisulfite converted genomic DNA. Illumina sequencing was used to sequence the fragments eluted from the arrays. The padlock capture strategy was developed by different groups for DNA methylation analysis in target regions [60,63]. Padlock probes were designed to capture the bisulfite converted targeted sequences. Then, they are ligated to form a circularized single strand of DNA in the target region, which can be further amplified and sequenced. Thousands of probes can be designed for the targeted regions like CGIs and the captured fragments can be sequenced in a single run by Illumina Genome Analyzer. For both above mentioned technologies, the capture efficiency of the designed probes, can potentially affect the measurement of DNA methylation state.

4) Illumina sequencing of bisulfite converted DNA was also used to quantify the DNA methylation level in mouse primordial germ cells at lower coverage that allowed to analyse some global methylation properties like a strong global reduction of DNA methylation in primordial germ cells [26].

### 2.3. Other NGS sequencing based DNA methylation analysis

SOLiD ^TM^ developed by Applied Biosyetems has been available since 2007. It is a short-read sequencing technology based on ligation. The sample preparation is similar to 454 sequencing, which is also based on emulsion PCR. DNA ligase, rather than polymerase, is used for sequencing the amplified fragments from single molecules. It is not as widely used as the above mentioned NGS sequencing method for DNA methylation analysis. Recently, Bormann Chung *et al.* reported the first whole methylome bisulfite sequencing study using SOLiD ^TM^ platform [64] in *E. coli*. HeliScope from Helicos BioSciences was the first single-molecular sequencing platform available since 2007. The biggest difference between this method and above mentioned NGS methods is in the templates prepared for sequencing. The templates of 454 sequencing, Illumina Solexa sequencing or SOLiD ^TM^ are all clonally amplified from single DNA molecules, while HeliScope directly uses the single DNA molecule as the template, which simplifies the sample preparation process, decreases the cost and avoids the possible bias introduced by the amplification. So far, this platform has not been widely used for DNA methylation analysis.

## 3. Discussion

During the past decade, DNA methylation analysis has undergone a major technological revolution. The recently developed NGS methods in particular when coupled to bisulfite conversion enabled researcher to conduct genome wide DNA methylation analysis in high throughput at single base resolution with high speed. In the following we discuss some issues related with data production and analysis that are of particular interest for NGS based bisulfite DNA methylation analysis.

### 3.1. Statistical issues in bisulfite sequencing DNA methylation analysis

**Figure 3:**
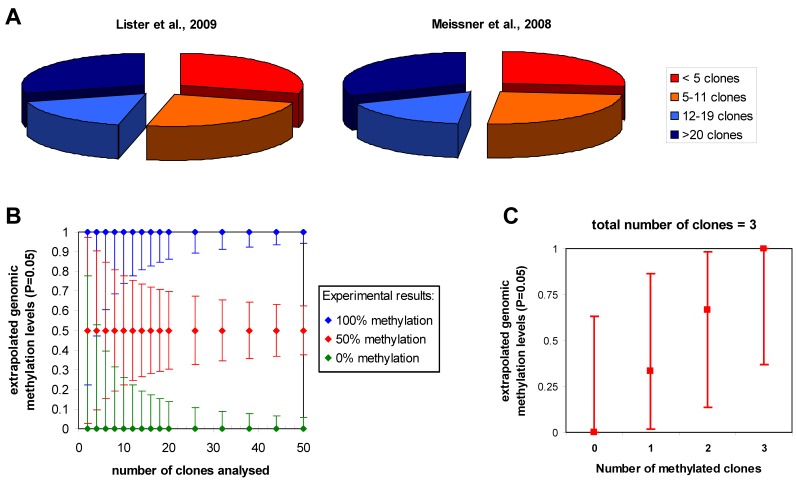
Statistics in bisulfite-seq DNA methylation analysis. **(a)** Sequence depth of the data sets provided by Meissner *et al.* (2008) and Lister *et al.* (2009). The pie diagram displays the fractions of all CpG sites for which less than 5 (red), 5 to 11 (orange), 12 to 19 (light blue) or more than 20 clones (dark blue) were available. **(b)** Confidence intervals for the extrapolation of genomic methylation levels from bisulfite sequencing calculated for experimental results assuming that all reads are methylated at one site (blue), 50% of the reads are methylated (red) or all reads are unmethylated (green). Depending on the number of reads the uncertainty of the extrapolation of the genomic methylation levels is getting smaller. Note, the high uncertainty with low sequencing depth. **(c)** Ambiguity of the extrapolation of genomic methylation levels illustrated using all possible results that could be obtained after analysis of 3 reads as an example.

An important issue to be considered is the influence of statistics on the accuracy of the estimation of the genomic methylation level from bisulfite sequencing data. Since the methylation level of a particular cytosine in the sample is extrapolated from the number of times a C or T is observed in the sequenced clones or sequencing reads at the corresponding position (called reads from now on), binomial statistics can be applied. Confidence intervals for methylation levels can be calculated for any experimentally observed number of cytosine and thymidine reads using exact binomial testing. In this approach, the probability of observing the experimental result is calculated for various theoretical genomic methylation levels. Then, the upper and lower limits of genomic methylation compatible with the experimental data at a certain level of stringency (like P values > 0.05) can be used to define the confidence intervals (Figure 3). As expected, the accuracy of this extrapolation increases with the read depth of the particular cytosine residue. Importantly, the uncertainties of the extrapolation of the true genomic methylation level are quite large with small number of reads. For example, with less than 5 reads one cannot even distinguish the methylation state of two sites even if all reads are methylated at one site and unmethylated at the other, because a genomic methylation level of 50% is compatible with both results. Hence, less than 5 reads provide little information on the DNA methylation of a particular CpG site in the sample. A minimum of 12 reads is needed to differentiate sites showing 50% methylation in one sample and 0 or 100% in another and at least 20 reads are needed to have error margins of the estimation of the genomic methylation level that are smaller than ±20% (if 50% methylation is experimentally observed). In cases of lower sequence depth, information on the average methylation of genomic loci may be obtained by combining adjacent methylation sites, but this will average potential site specific patterns and no longer provide single site resolution.

To give some examples, the genome wide reduced representation bisulfite sequencing studies of Meissner *et al.* (2008) [21] and the genome wide analysis of Lister *et al.* (2009) [15] provide detailed statistics about the number of reads at each cytosine which allows subdividing the data set into these categories (Figure 3b). The distributions look very similar for both studies: roughly one quarter of the cytosine residues had less than 5 reads, a second quarter had between 4 and 11 reads, a third quarter had 12 to 20 reads and for the remaining quarter of cytosines more than 20 reads were available. In a combined array-based hybrid selection and bisulfite sequencing approach Hodges *et al.* achieved >92% of the target regions with >10 reads [62]. Taking into account the sequencing depth and the results, the authors use binomial statistics to define three categories of methylation levels (unmethylated, partially methylated and methylated). In contrast, Laurent *et al.* (2010) assume that already 3 reads would be enough to call methylation levels with sufficient confidence [16]. This assumption is not correct as illustrated in Figure 3, which shows that all potential results that could be obtained with 3 reads (*i.e.*, zero, 1, 2, or 3 methylated cytosines) are compatible with genomic methylation levels between 37% and 63%. In summary, it will be desirable to improve sequence depth about tenfold in future studies to allow for detailed comparisons of methylation patterns from different biological samples at single cytosine resolution. Given the current pace in the development of sequencing technology, this goal certainly is within reach.

### 3.2. Methylation analysis in repeats

The methylation state of CpG sites in repetitive sequences is still hard to analyze. The reason for this is that repeats may be present in 10000-100000 copies in the genome, which all are very similar in sequence. For methods based on bisulfite conversion the situation is even worse, because all unmethylated cytosines are converted to thymine, resulting an even lower complexity of the sequence. Therefore, for repetitive sequences, an alignment of the reads to the genomic sequence is only possible, if the read includes unique sequences outside of the repeat. It is estimated that approximately 1/10 of the CpG sites in the mammalian genome will be hard to align [11] after bisulfite conversion. Longer sequence reads are expected to get more accurate alignment and increase the genome coverage of the dataset.

### 3.3. Clonal DNA amplification after bisulfite conversion

One important caveat of bisulfite methylation analysis is the possibility of amplifying single converted DNA molecules (“clonal PCR”), which after subcloning of the PCR product and sequencing of individual clones, could give rise to several identical sequences which will bias the result. Similar clonal amplification of DNA after bisulfite conversion can happen if bisulfite conversion is coupled to NGS and led to several reads all representing the same original DNA molecule. After clonal amplification, the data will pretend a sufficient sequencing depth, which is a misinterpretation if all sequence reads relate to one individual original molecule of DNA. In conventional bisulfite studies, analysis softwares often attempt to filter for clonal sequences by considering clones with identical methylation pattern or clones with identical pattern of incomplete conversion and identical methylation pattern as clonal. However, this filter can never be fully reliable and, in principle, results need to be confirmed by independent conversion of the same template DNA. NGS methods provide a straightforward solution to this general and very important problem in bisulfite DNA methylation analysis, since they always include a ligation step of linkers or adaptors to the DNA fragments which happens before the first PCR amplification. These linkers can contain some randomized positions (“barcode”), which will later allow to discriminate if different reads were amplified from the same original DNA template [40,65]. So far, barcodes with fixed sequences in the adaptors have been employed for the sequencing and analysis of multiple samples in one sequencing run [66,67]. Adding some randomized nucleotides to these adaptors is highly recommended to allow for straightforward filtering of clonal reads and should be a regular step in NGS bisulfite DNA methylation analyses. The HeliScope platform and several novel NGS platforms under development, which are based on single-molecule sequencing technology without applying a PCR amplification, can avoid such problems as well.

### 3.4. Detection of non-CpG methylation

Another problem of bisulfite DNA methylation analysis is the incomplete conversion of cytosine, which cannot be discriminated from methylation. Modern protocols of bisulfite treatment can reach high conversion rates on purified DNA [68]. However, since conversion only happens on single stranded DNA, its efficiency is influenced by the DNA preparation. For example, contamination with DNA binding protein like histones will interfere with DNA denaturation and thereby lower conversion efficiency. In addition, the DNA sequence may also affect conversion efficiency, because stable secondary structure elements forming in the DNA after denaturation also interfere with conversion. In the study of mammalian DNA methylation patterns where methylation basically happens only in CpG sites, the cytosine residues observed at non-CpG sites are often taken as an indicator for conversion efficiency [69]. However, this approach is not feasible for plant and fungi DNA, which both show methylation also at non-CpG sites. Recently, non-CpG methylation has been reported to occur as well in human embryonic stem cells: Lister *et al.* report 0.02% non-CpG methylation in fetal lung fibroblasts and 24.5% in human embryonic stem cells [15]. In contrast, Laurent *et al.*, report 15% non-CpG methylation in fetal foreskin fibroblasts and 20% embryonic stem cells [16]. Different approaches can be used to discriminate between non-CpG methylation and incomplete conversion:

1. Analysis of mitochondrial DNA which is not methylated can be included for conversion control. However, this is not bound to chromatin and the DNA sequence is different so it may not be sufficient as control.
2. Sequencing depths can be increased. If non-CpG methylation is happening and biologically relevant, it should be observed in several reads at the same cytosine residue. It is essential at the same time to use barcoded adaptors to exclude clonal amplification of the DNA templates giving rise to the independent reads with same non-CpG methylation patterns.
3. Results can be reproduced with independent DNA preparations.
4. In order to avoid conversion problems related to the primary sequence, recombinant DNA with same sequence can be added to the genomic DNA and analyzed.
5. At key positions, methylation may be confirmed by methods not based on bisulfite conversion.

### 3.5. The challenge of data analysis

The huge amount of data generated by the NGS platforms in the form of short reads, presents another challenge for the developing of more efficient software and computer algorithms for sequence alignment, base calling, and statistical analysis. Some softwares and bioinformatics tools have been developed for the data analysis, as summarized in [70]. However, the methods on accurate alignment of the reads to the unique genomic locations after bisulfite conversion, the DNA methylation percentage determination and data presentation and deposition still need to be optimized.

In summary, the available NGS methods make it possible to obtain quantitative DNA methylation data at single base pair resolution and with genome wide coverage. They will greatly facilitate the profiling of the DNA methylation in the genomes of different species, individuals and cell types under healthy and disease states. So far, several novel NGS method are under development, e.g. VisiGen, which is a platform based on real time single-molecule sequencing nanosequencing technology, the single-molecule nanopore DNA sequencing platform [71], which can distinguish the methylated cytosine from the four standard DNA bases directly without bisulfite pretreatment. The Pacific Biosciences platform is based on single molecule real-time (SMRT) technology and can directly detect methylated DNA including N6-methyladenine and 5-methylcytosine without bisulfite conversion as well [72]. The SMRT sequencing can also detect the 5hmC, which cannot be distinguished from methylated cytosine using methods based on bisulfite conversion. These novel NGS platforms have advantages in the less bias during template preparation, possible longer read length, lower cost, higher speed and better accuracy. We are expecting to see how DNA methylation profiling will benefit from these novel NGS platforms, 
